# Prolonged Diagnostic Intervals as Marker of Missed Diagnostic Opportunities in Bladder and Kidney Cancer Patients with Alarm Features: A Longitudinal Linked Data Study

**DOI:** 10.3390/cancers13010156

**Published:** 2021-01-05

**Authors:** Yin Zhou, Fiona M. Walter, Hardeep Singh, William Hamilton, Gary A. Abel, Georgios Lyratzopoulos

**Affiliations:** 1 Primary Care Unit, Department of Public Health and Primary Care, University of Cambridge, Worts’ Causeway, Cambridge CB1 8RN, UK; fmw22@medschl.cam.ac.uk; 2 Center for Innovations in Quality, Effectiveness and Safety, Michael E. DeBakey Veterans Affairs Medical Center and Baylor College of Medicine, Houston, TX 77030, USA; hardeeps@bcm.edu; 3 College of Medicine and Health, University of Exeter Medical School (Primary Care), Exeter EX1 1TX, UK; W.Hamilton@exeter.ac.uk (W.H.); g.a.abel@exeter.ac.uk (G.A.A.); 4 Epidemiology of Cancer Healthcare and Outcomes (ECHO) Research Group, Department of Behavioural Science and Health, University College London, London WC1E 6BT, UK; y.lyratzopoulos@ucl.ac.uk

**Keywords:** bladder cancer, kidney cancer, early diagnosis, diagnostic timeliness, missed opportunity

## Abstract

**Simple Summary:**

In England, patients with alarm features of cancer should be assessed by a specialist within 14 days based on national guidelines. However, it is not known how quickly these patients are actually diagnosed. We therefore examined how quickly patients who met these fast-track referral criteria were actually diagnosed, using bladder and kidney cancer patients as exemplars. We found that of the patients who qualified for fast-track referral, more than one-quarter did not receive a timely diagnosis. Those with recurrent urinary tract infections, of female sex and in the extremes of age, were most likely to have a non-timely diagnosis. Our findings suggest that opportunities exist to improve timely referral in patients with bladder and kidney cancer.

**Abstract:**

Background: In England, patients who meet National Institute for Health and Care Excellence (NICE) guideline criteria for suspected cancer should receive a specialist assessment within 14 days. We examined how quickly bladder and kidney cancer patients who met fast-track referral criteria were actually diagnosed. Methods: We used linked primary care and cancer registration data on bladder and kidney cancer patients who met fast-track referral criteria and examined the time from their first presentation with alarm features to diagnosis. Using logistic regression we examined factors most likely to be associated with non-timely diagnosis (defined as intervals exceeding 90 days), adjusting for age, sex and cancer type, positing that such occurrences represent missed opportunity for timely referral, possibly due to sub-optimal guideline adherence. Results: 28%, 42% and 31% of all urological cancer patients reported no, one or two or more relevant symptoms respectively in the year before diagnosis. Of the 2105 patients with alarm features warranting fast-track assessment, 1373 (65%) presented with unexplained haematuria, 382 (18%) with recurrent urinary tract infections (UTIs), 303 (14%) with visible haematuria, and 45 (2%) with an abdominal mass. 27% overall, and 24%, 45%, 18% and 27% of each group respectively, had a non-timely diagnosis. Presentation with recurrent UTI was associated with longest median diagnostic interval (median 83 days, IQR 43–151) and visible haematuria with the shortest (median 50 days, IQR 30–79). After adjustment, presentation with recurrent UTIs, being in the youngest or oldest age group, female sex, and diagnosis of kidney and upper tract urothelial cancer, were associated with greater odds of non-timely diagnosis. Conclusion: More than a quarter of patients presenting with fast-track referral features did not achieve a timely diagnosis, suggesting inadequate guideline adherence for some patients. The findings highlight a substantial number of opportunities for expediting the diagnosis of patients with bladder or kidney cancers.

## 1. Background

Timeliness has been regarded as one of the important domains of high quality health care [1], and is particularly relevant for evaluating the diagnostic process in cancer [2]. Timely diagnosis of cancer is crucial for improving clinical and patient-reported outcomes [3,4]. Bladder and kidney cancers, with 5-year survival rates of about 53% and 64% in England respectively [5], can be challenging to diagnose as about a third of patients with bladder and three quarters of patients with kidney cancer present without haematuria [6]. Urinary tract infections (UTIs) and benign prostatic conditions are common and can both mimic and co-occur with underlying cancer [7,8].

Timely referral to secondary care for suspected cases is important, given that the majority of urological cancers are diagnosed following a referral by a general practitioner (GP) in England [9,10]. The National Institute of Clinical Excellence (NICE) has produced guidelines to help identify patients with high risk of cancer based on the presence of alarm symptoms and signs (clinical features which carry a positive predictive value (PPV) for cancer of 3% or greater in the 2015 guidelines, and 5% of greater in the 2005 guidelines) [11] in order to improve timely diagnosis of cancer. Patients with clinical features that meet the NICE guidelines ought to be referred by their GPs on a fast-track system (the two-week-wait pathway), to be assessed by a specialist within two weeks. Despite the existence of these guidelines since 2005, with an update in 2015, timely diagnosis may be challenging due to system, clinical or patient factors, with large variations observed in the odds of fast-track referrals by cancer [12]. Given that women with urological cancer in general experience a longer time to diagnosis than men [8] and are more likely to be diagnosed through an emergency presentation [13], we were interested to find out whether this gender inequality existed even among patients of either sex with the same presenting features which met fast-track referral criteria. Despite evidence of diagnostic delay in patients with other cancers who presented with alarm features [14,15], little is known about the timeliness of diagnosis in patients with potential bladder and kidney cancer who fulfil the fast-track referral criteria, and whether sub-optimal guideline adherence could be a marker for missed opportunity in achieving a timely diagnosis.

In this study, we examined the frequency of relevant clinical features and diagnostic timeliness among patients diagnosed with bladder and kidney cancer, focusing on patients who presented with alarm features warranting a fast-track referral. We examined whether these patients received a diagnosis reasonably soon after a presentation meriting prompt specialist assessment, and factors that predict increased risk of longer interval from presentation to diagnosis, positing that non-timely diagnosis in these cases may signal missed opportunities for timely referral.

## 2. Method

### 2.1. Data

Linked data from primary care records (Clinical Practice Research Datalink, CPRD) and Cancer Registry were obtained for a series of studies, with details of the data extraction process and cancer cohort identification previously described [16]. Patients aged 25 and above who were diagnosed with bladder and kidney cancer between April 2012 and December 2015 were extracted from the CPRD. These data were linked at source to the Cancer Registry, from which additional cases were identified using ICD-10 cancer codes. We used the Cancer Registry diagnosis and date where available, and CPRD diagnosis and date in patients without linked data. Cancers were sub-divided into bladder, kidney or upper urinary tract urothelial cell cancer (UUTUCC, subsequently referred to as upper tract urothelial cancer, or UTUC).

Symptoms, signs and diagnoses were extracted from coded information within CPRD (Table S1 for code lists). As our data comprised patients diagnosed before or just after the newly updated NICE guidelines in 2015, we used the 2005 NICE guidelines CG27 as the basis for creating scenario groups of patients who met the fast-track referral guidelines, using operational definitions (detailed below, and in Table 1) agreed by all clinical co-authors (YZ, FMW, HS, WH, GL), three of whom are GPs in England. Demographic variables including age and sex were obtained from CPRD.

This work uses data provided by patients and collected by the National Health Service as part of their care and support. A study protocol (17_107R) was submitted to and approved by CPRD prior to study commencement. Ethical review and approval were waived for this study, due to the data being provided to researchers in an anonmyised form, and individual consent not required.

### 2.2. Defining Fast-Track Scenarios

Five clinical scenarios were initially created based on the four key recommendations from the fast-track guidelines for suspected bladder or kidney cancer (Table 1). Patients who qualified for the fast-track referral criteria were regarded as “scenario positive”.

Defining ‘haematuria’ was complicated by the high number of patients with non-specific haematuria codes (*n* = 1679 for unspecified, 421 for visible haematuria and 299 for non-visible haematuria). Because the investigative pattern and diagnostic timeliness could differ across the haematuria types, we considered patients with a non-specific haematuria code separately instead of assuming visible or non-visible. Once the clinical presentation scenarios were defined, we found that only 17 patients with non-visible haematuria fulfilled fast-track criteria; they were therefore excluded from further analysis due to the small number of patients, resulting in a total of four remaining scenarios for analysis. The remaining group of patients, with visible and unspecified haematuria, formed scenarios 1 and 2 respectively (Table 1). Using similar approaches based on existing literature [17], we excluded patients with record of diagnosis of either UTI or bladder/kidney-lithiasis recorded within 30 days pre- and post- the haematuria episode to exclude haematuria due to an acute cause. Diagnostic interval was measured from the haematuria record to cancer diagnosis.

Recurrent UTIs (scenario 3) was defined as either two episodes of UTI within 6 months or three within 12 months [18], with the diagnostic interval being measured from the second or third UTI, respectively, to cancer diagnosis. At least one haematuria event during the qualifying period was also required to be regarded as “scenario positive” for having recurrent UTIs, concordant with the fast-track guidelines which included those “aged 40+ with recurrent or persistent UTI associated with haematuria” (Table 1).

Patients with a recorded abdominal mass were included in scenario 4. Diagnostic intervals were calculated from the date of this code being recorded in the patient’s record to the cancer diagnosis.

In patients who qualified for two or more scenarios, we regarded the first scenario as the index scenario at which a fast-track referral could be expected.

### 2.3. Diagnostic Timeliness

We examined diagnostic timeliness for patients with one of the four studied presentation scenarios, by calculating the median and the accompanying interquartile range for the number of days from the primary care consultation at which the patient first qualified for a fast-track referral to cancer diagnosis (which we termed the NICE-diagnostic interval, NICE-DI).

There is evidence that patients with certain cancers (including bladder cancer) who are diagnosed more than 3 months after initial presentation have poorer survival rates than those diagnosed within 3 months [19,20]. We therefore defined a timely diagnosis as one that was made up to and including 90 days of meeting the referral criteria, which was also in line with previous similar studies [21,22]. Given that a fast-track referral enables a patient to be assessed by a specialist and subsequent investigations to be initiated within 14 days of a GP referral, we considered our 90-day threshold for defining timeliness to be a conservative estimate. In cases where the NICE-DI was beyond 90 days, we postulated that probable missed opportunity for timely referral existed, likely resulting from deviation from guideline recommended care.

### 2.4. Analysis

We first described the frequency of relevant clinical features present in all patients reported in the year before cancer diagnosis, and the number of patients who met each of the four scenarios where fast-track specialist assessment is recommended. We then reported the median and interquartile ranges for the NICE-DI in patients who were “scenario positive” by measuring the interval from when they qualified for fast-track referral to diagnosis (Table 1).

We used logistic regression to examine the factors that were associated with non-timely diagnosis (as above, defined as having a NICE-DI of more than 90 days), first crudely, then adjusting for cancer site, presenting clinical scenario, age and sex. Among patients with observed data we found no evidence for variation by deprivation group or ethnicity, and therefore did not include these variables in subsequent analyses. Socio-demographic variables were chosen as covariates due to prior evidence of their associations with odds of fast-track referral [12]. We then repeated the adjusted analysis using different cut-offs of the NICE-DI, 45 and 60 days respectively, to see if the associations remained between the explanatory and outcome variables.

All analyses were performed using Stata version 15 (StataCorp LLC., Allen, TX, USA)

## 3. Results

### 3.1. Clinical Features

5319 patients with urological cancer were examined for relevant clinical features pre-diagnosis (Table 2). 1464 patients (28%) had no relevant clinical features recorded in the 12 months pre-diagnosis. Of the 3855 patients with one or more relevant features (Table S2), the mean number of recorded symptoms was 1.6: 58% had one recorded feature (higher proportion of bladder than kidney cancer patients), and 42% had two or more clinical features (Table 2).

Almost two-third of bladder cancer patients and 40% of upper tract urothelial cancer patients had at least one episode of haematuria, while about 16% of kidney cancer patients had haematuria. The symptom signature of bladder cancer was dominated by haematuria, while that of kidney cancer was broader, with similar proportions of people presenting with haematuria, UTI and abdominal pain (between 13–16%). (Table 2).

### 3.2. Patients Who Met Fast-Track Referral Criteria

2105 patients met one of the fast-track referral criteria and were included in subsequent analyses. Their distribution was as following: Bladder cancer (*N* = 1713, 81%), kidney cancer (*N* = 311, 15%) and upper tract urothelial cancer (*N* = 81, 4%). Of all patients with bladder and kidney cancer in our original sample, the most common fast-track scenario was unspecified haematuria (Scenario 1, 25.8%) and the least common (only 0.8%) was abdominal mass (Scenario 4) (Table 3).

Patients with recurrent UTIs had the longest NICE-DI, with a median duration of 83 days (IQR 43-151); this contrasted with those with visible haematuria, in whom the median NICE-DI was the shortest at 50 days (IQR 30-79). Relatedly, patients with recurrent UTIs were the most likely, while those with visible haematuria were the least likely, to have a non-timely diagnosis. Around a fifth to a quarter of patients with visible haematuria, unspecified haematuria and abdominal mass, (18%, 24% and 27% respectively) and almost half (45%) of those with recurrent UTIs received a non-timely diagnosis.

The highest proportions of patients across all clinical scenarios were between 65 and 84 years of age. There was a higher proportion of men compared to women in those with visible haematuria (78%), unspecified haematuria (78%) and abdominal mass (56%), and lower proportion in those with recurrent UTIs (48%).

### 3.3. Factors Associated with Non-Timely Diagnosis

There was a similar direction of associations between cancer site, scenario and sex, with non-timely diagnosis in the crude and adjusted models, with kidney and UTUC cancer, scenarios other than visible haematuria, and female sex predicting higher odds of a non-timely diagnosis (Table 4). However, the effect size for sex was smaller in the adjusted than crude models. This was largely due to higher proportions of recurrent UTIs in women (34% compared with 12% in men), and to a smaller extent, of kidney cancer in women (18% compared with 14% in men). In both crude and adjusted models, patients with recurrent UTIs had the highest odds of having a non-timely diagnosis (adjusted OR 3.46, CI 2.40–5.00; *p* < 0.0001).

Considering the adjusted model, patients subsequently diagnosed with kidney cancer who met fast-track referral criteria had higher odds of having a non-timely diagnosis than those with bladder cancer (adjusted OR 2.00, CI 1.52–2.63, *p* < 0.0001). Patients with recurrent UTIs had almost 3.5 times and those with unspecified haematuria 1.5 times greater odds of having a non-timely diagnosis, compared to people with visible haematuria who met fast-track criteria (adjusted OR 1.43, CI 1.04–1.98 for unspecified vs. visible haematuria, *p* < 0.0001). Compared to men, women had greater odds of having a non-timely diagnosis (adjusted OR 1.33, CI 1.09–1.66, *p* = 0.011). When applying more stringent cut-offs for timely diagnosis (45 and 60 days), the overall patterns of association between exposure and outcome variables remained the same for each model, although the effect of sex was greatest and strongest when considering a longer delay (90-day cut-off) (in Table S3).

There was an inverse J-shaped association between age groups and having a non-timely diagnosis, with the youngest and oldest patients having the highest odds (*p* = 0.047).

## 4. Discussion

About a quarter of patients with bladder and kidney cancer who had presented with features which warranted a fast-track referral did not receive a timely diagnosis, and such occurrences were likely to represent missed opportunity for timely referral due to sub-optimal guideline adherence. Those with guideline-recommended fast-track referral for recurrent UTIs associated with haematuria had the longest NICE-DI while those with visible haematuria the shortest. Nearly one in five of those with visible haematuria and half of all patients with recurrent UTIs had a non-timely diagnosis. In adjusted analysis, patients with recurrent UTIs, women, and patients at the extremes of ages were most likely to have a non-timely diagnosis, as were patients subsequently found to have kidney cancer.

### 4.1. Strengths and Limitations

To our knowledge this is the first study to use a large representative cohort to study how often bladder and kidney cancer patients warranting a fast-track referral are diagnosed in a non-timely fashion, as a marker of clinician guidelines adherence in patients who qualify for guideline recommended specialist diagnostic assessment. Despite challenges with identifying signals of missed diagnostic opportunities using large electronic health records [23], we were able to develop clinically adjudicated scenarios that captured the recurrence and unexplained nature of clinical features. Our findings highlight the patient groups who were most at risk of having a non-timely GP referral, so that we can better understand how and why non-timely diagnosis of bladder and kidney cancer might occur. Similar methods could be applied to diagnosis of other cancers.

Only coded information on presenting clinical features was available from CPRD, which might lead to both under- and over-estimation of the true effects of presenting clinical features. A previous study found that about 37% of visible haematuria records in bladder cancer patients were within hidden text in CPRD [24]. Given the possibility of variation in coding behaviour in clinical practice, all chosen variables and scenarios were considered and refined by clinician co-authors to ensure that the operational definitions were as representative and valid as possible. Furthermore, our study objective was to examine the patterns of variation instead of measuring an outcome measure of these clinical features, and therefore the data can still be informative for our purpose of enquiry.

### 4.2. Implications

Consistent with evidence showing patients presenting with UTI were likely to have a longer time to diagnosis [8,22], we found that patients with recurrent UTIs who qualified for fast-track referral were also the most likely to have a longer time to diagnosis than those with other alarm clinical features. Diagnostic reasoning for possible urological cancer may be particularly challenging when a UTI is present as it can represent a concomitant unrelated condition or similar symptoms be related to the underlying cancer, for example, due to irritation or urinary stasis [25]. Nevertheless, our findings imply that this group of patients was less likely to receive guideline concordant care than patients with other alarm features.

Patients with unspecified haematuria (with visible and non-visible haematuria in unknown proportions) had a longer diagnostic interval and 1.5 times greater odds of having a non-timely diagnosis than those with visible haematuria. This is likely to reflect lower positive predictive value of non-visible haematuria for cancer compared to the other fast-track qualifying scenarios [26], which may result in clinicians not acting swiftly or not adhering to guidelines when the chances of cancer are smaller [10]. The updated 2015 NICE guidelines and the 2020 American Urological Association include additional clinical criteria to further risk stratify patients with non-visible haematuria, in order to increase the diagnostic yield of cancer in the referred population [11,27]. Clinical decision aids such as risk prediction tools incorporating multiple biological and patient risk factors may help in situations where diagnostic reasoning is challenging, such as by identifying patients with non-visible haematuria and recurrent UTIs who are at higher risk of bladder cancer [28,29].

Women meeting fast-track criteria had higher odds of having a non-timely diagnosis than men. Assuming that fast-track referrals resulted in similar secondary care intervals (from referral to diagnosis), it is likely that the timeliness of the primary to secondary care referral was the main contributor to the sex difference seen in the overall NICE-DI. Women are more likely to have 3 or more pre-referral consultations than men before their urological cancer diagnosis, supporting this argument [30]. Women’s predisposition to UTIs may play a role in them being referred and diagnosed later than men [8]. Indeed we found that around half of the sex difference (which was more pronounced among patients with the longest delays) could be explained by adjustments for presenting features. A further explanation for lower guideline adherence in women than men is that primary care clinicians may be conscious that the risk of urological cancer in women is lower than men, even when women and men present with the same symptoms, resulting in differing thresholds for referral.

Our study highlighted that despite meeting fast-track referral guideline criteria (with which positive predictive value for cancer was of 5% or greater), some patients were disadvantaged with respect to having a timely referral. Rigid consultation norms (such as short consultation duration), suboptimal history taking and examination, language barriers, clinician cognitive biases, and health system constraints have been found to contribute to missed diagnostic opportunities in the initial assessment and referral of cancer patients [31,32,33].

Measurement methodologies that identify signals for potential missed opportunities, such as the one described here, can provide the data needed to guide improvement. Use of electronic data, including information available in clinical notes, can help identify situations where action is needed on alarm features [34]. For instance, in-consultation electronic triggers highlighting men and women who meet referral criteria, as well as post-consultation triggers flagging up patients with alarm features but who have no subsequent investigative or referral actions may serve as helpful reminders to GPs to consider further investigations, during and after the index consultation, in order to reduce missed diagnostic opportunities [35,36]. Post-consultation electronic triggers based on algorithms could identify and reduce missed follow-up of cases resulting from patient, clinician or system factors that lead to the failure of closure of the diagnostic loop (for example, when a requested test or referral has not taken place because of administrative delays or patient no-show) [2,34,37].

Although our findings highlighted inequalities in cancer patients who were at risk of having a non-timely referral, it is possible that missed diagnostic opportunities due to a missed or non-timely referral could also occur in patients subsequently diagnosed with disease other than cancer. Further population-based research to determine which symptomatic patients are at risk of having a non-timely referral, complemented by in-depth record review and/or qualitative studies to explore how and why they occur, are paramount [23].

## 5. Conclusions

At least one in four patients with bladder and kidney cancer with alarm symptoms were diagnosed more than 90 days after meeting fast-track referral guidelines. Patients with kidney cancer, and women with recurrent UTIs were most likely to have a non-timely diagnosis, possibly as a result of a delayed referral due to guideline deviation. The co-presence of benign and malignant disease (e.g., recurrent UTI as a genuine presenting feature of bladder cancer) poses challenges for diagnostic reasoning. Measurement of presentation to diagnosis intervals using routinely available electronic health record data can be a promising avenue for health system-wide monitoring of diagnostic quality and safety. Risk prediction tools, in-consultation alerts and post-consultation triggers may help clinicians minimise some of the potential contributors to a non-timely referral in patients with urological alarm symptoms, paving an important step towards reducing the overall sex inequality seen in diagnostic timeliness of urological cancer patients.

## Figures and Tables

**Table 1:** Operational definitions for the fast-track scenarios as per clinical guidelines.

Scenario	Fast-Track Referral Criteria as Per NICE Guidelines	Schematic Representation of Operational Definitions for Each Scenario	Definition of NICE- Diagnostic Interval (NICE-DI)
1 Visible haematuria	Painless macroscopic haematuria	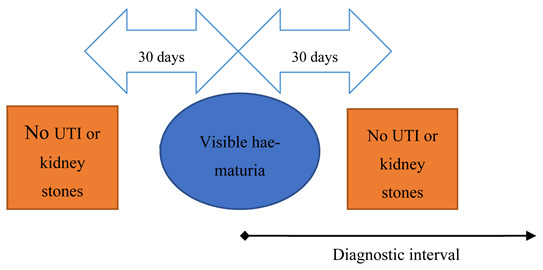	From episode of visible haematuria to cancer diagnosis.
2 Unspecified haematuria (non-visible haematuria also initially considered but excluded due to small numbers)	Aged 50+ with unexplained microscopic haematuria	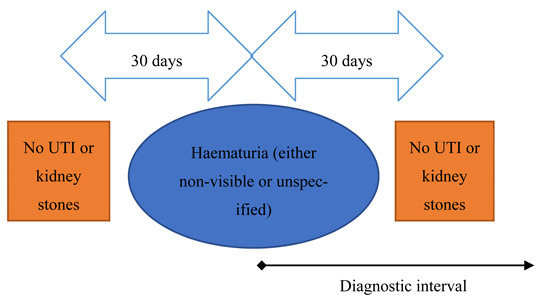	From episode of haematuria to cancer diagnosis.
3 Recurrent urinary tract infections (UTIs)	Aged 40+ with recurrent or persistent UTI associated with haematuria	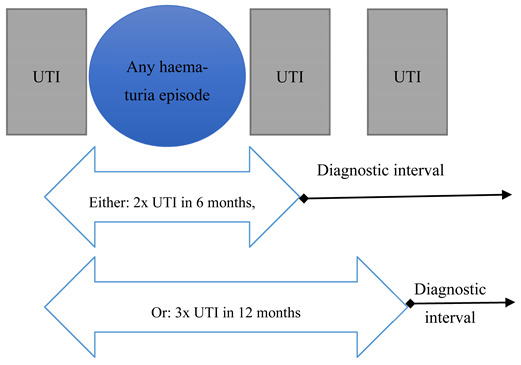	From second UTI (in 6 months) or third UTI (in 12 months), to cancer diagnosis.
4 Abdominal mass	Abdominal mass identified clinically or on imaging that is thought to be arising from the urinary tract.	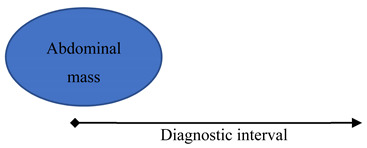	From recorded abdominal mass to cancer diagnosis.

**Table 2:** Number of patients with recorded relevant clinical features in the 12 months pre-diagnosis.

Clinical Feature ^	All Patients N = 5319		Bladder Cancer N = 3397		Kidney Cancer N = 1714		UTUC N = 208	
	N	*%*	N	*%*	N	*%*	N	*%*
**Number of features**								
None	1464	27.5	755	22.2	649	37.9	60	28.8
One	2231	41.9	1518	44.7	637	37.2	76	36.5
Two or more	1624	30.5	1124	33.1	428	25.0	72	34.6
Alarm clinical features								
Haematuria	2137	40.2	1773 *	61.2 *	279 *	16.3 *	85 *	40.9 *
Urinary tract infection ^^	1149	21.6	885 *	30.6 *	217 *	12.7 *	47 *	22.8 *
Abdominal mass	50	0.9	11	0.4	37	2.2	2	1.0
**Other urinary symptoms**								
Nocturia	101	1.9	74	2.6	26	1.5	1	0.5
Poor stream	18	0.3	15	0.5	0	-	3	1.4
Hesitancy	0	-	-	-	-	-	-	-
Urinary retention	90	1.7	63	2.2	25	1.5	2	1.0
Urinary incontinence	67	1.3	50	1.7	16	0.9	1	0.5
**Pain**								
Kidney stone/loin pain	187	3.5	77	2.7	96	5.6	14	6.7
Biliary colic	10	0.2	2	0.1	8	0.5	0	-
Pelvic pain	83	1.6	45	1.6	32	1.9	6	2.9
Abdominal pain	500	9.4	250 *	8.6 *	226 *	13.2 *	24 *	11.5 *
Low back pain	408	7.7	220	7.6	168	9.8	20	9.6
**Others**								
Abd. distension	15	0.3	8	0.3	6	0.4	1	0.5
Fatigue	178	3.3	85	2.9	85	5.0	8	3.8
Weight loss	131	2.5	61	2.1	62	3.6	8	3.8
Loss of appetite	28	0.5	11	0.4	16	0.9	1	0.5
Fever/night sweats	47	0.9	21	0.7	24	1.4	2	1.0
Anaemia **	219	4.1	58	2.0	50	2.9	6	2.9

UTUC: Upper tract urothelial cancer. * Top three most frequent clinical features for each cancer type. ** Clinical codes of anaemia included only, not including lab results for low haemoglobin. ^ Patients with more than one clinical feature coded are represented in all of their relevant features (i.e., multiple rows within table). ^^ UTI consists of codes for UTI, dysuria, urinary frequency, urinary urgency and malodourous urine (see S1 for full list of codes).

**Table 3:** Frequency of patients by fast-track scenario types, stratified by cancer site, age and sex.

			**Number of Patients (%) for Each Scenario**		
	All Scenarios (*n* = 2105, 39.6% *)	Visible Haematuria (*n* = 305, 5.7% *)	Unspecified Haematuria (*n* = 1373, 25.8% *)	Recurrent UTIs (*n* = 382, 7.2% *)	Abdominal Mass (*n* = 45, 0.8% *)
**Cancer Site**					
**Bladder**	1713 (81.4%)	264 (86.6%)	1129 (82.2%)	310 (81.2%)	10 (22.2%)
**Kidney**	311 (14.8%)	29 (9.5%)	194 (14.1%)	54 (14.1%)	34 (75.6%)
**UTUC**	81 (3.8%)	12 (3.9%)	50 (3.6%)	18 (4.7%)	1 (2.2%)
**Time to diagnosis**					
**Days (IQR)**	55 (34–95)	50 (30–79)	52 (33–89)	83 (43–151)	60 (26–92)
**Diagnosis beyond**					
**90 days**	571 (27.1%)	54 (17.7%)	332 (24.2%)	173 (45.3%)	12 (26.7%)
**Age groups**					
**<35**	7 (0.3%)	2 (0.7%)	5 (0.4%)	0 (0.0%)	0 (0.0%)
**35–44**	32 (1.5%)	5 (1.6%)	18 (1.3%)	9 (2.4%)	0 (0.0%)
**45–54**	141 (6.7%)	23 (7.5%)	85 (6.2%)	29 (7.6%)	4 (8.9%)
**55–64**	330 (15.7%)	51 (16.7%)	231 (16.8%)	39 (10.2%)	9 (20.0%)
**65–74**	688 (32.7%)	94 (30.8%)	463 (33.7%)	119 (31.2%)	12 (26.7%)
**75–84**	664 (31.5%)	94 (30.8%)	433 (31.5%)	123 (32.2%)	14 (31.1%)
**85+**	243 (11.5%)	36 (11.8%)	138 (10.1%)	63 (16.5%)	6 (13.3%)
**Sex**					
**Male**	1511 (71.8%)	237 (77.7%)	1068 (77.8%)	181 (47.4%)	25 (55.6%)
**Female**	594 (28.2%)	68 (22.3%)	293 (21.3%)	201 (52.6%)	20 (44.4%)

IQR: Interquartile range; UTIs: Urinary tract infections; UTUC: Upper tract urothelial cancer. * Percentages calculated against total cohort of 5319 patient.

**Table 4:** Association between scenario, patient and cancer characteristics and non-timely diagnosis.

	Number of Patients		Time to Diagnosis	Predictors of a Non-Timely Diagnosis, Odds Ratio (95% CI)			
	Total N	Non-Timely Diagnosis n (%)	Median Days (IQR)	Crude ORs	*p*-Value *	Adjusted ORs	*p*-Value *
**Cancer site**							
**Bladder**	1713	418 (24.4)	51 (33–90)	Reference	<0.0001	Reference	<0.0001
**Kidney**	311	115 (37.0)	70 (41–115)	1.82 (1.41, 2.35)		2.00 (1.52, 2.63)	
**UTUC**	81	38 (46.9)	85 (57–137)	2.74 (1.75, 4.29)		2.75 (1.72, 4.38)	
**Scenario**							
**Visible haematuria**	305	54 (17.7)	50 (30–79)	Reference	<0.0001	Reference	<0.0001
**Unspecified haematuria**	1373	173 (45.3)	52 (33–89)	1.48 (1.08, 2.04)		1.43 (1.04, 1.98)	
**Recurrent UTIs**	382	332 (24.2)	83 (43–151)	3.85 (2.69, 5.49)		3.46 (2.40, 5.00)	
**Abdominal mass**	45	12 (26.7)	60 (26–92)	1.69 (0.82, 3.48)		1.03 (0.49, 2.20)	
**Sex**							
**Male**	1511	365 (24.2)	52 (33–89)	Reference	<0.0001	Reference	0.0112
**Female**	594	206 (34.7)	65 (37–119)	1.67 (1.36, 2.05)		1.33 (1.07, 1.66)	
**Age group**							
**<35**	7	2 (28.6)	46 (22–93)	1.02 (0.20, 5.29)	0.1325	1.31 (0.24, 7.04)	0.0471
**35–44**	32	8 (25.0)	60.5 (42.5–90)	0.85 (0.37, 1.92)		0.75 (0.32, 1.73)	
**45–54**	141	25 (17.7)	52 (34–78)	0.55 (0.35, 0.87)		0.48 (0.30, 0.77)	
**55–64**	330	80 (24.2)	50 (30–88)	0.81 (0.60, 1.10)		0.83 (0.61, 1.14)	
**65–74**	688	194 (28.2)	56 (35–97)	Reference		Reference	
**75–84**	664	188 (28.3)	56 (34–101)	1.01 (0.79, 1.27)		1.05 (0.82, 1.34)	
**85+**	243	74 (30.5)	61 (38–126)	1.11 (0.81, 1.54)		1.06 (0.76, 1.48)	

CI: Confidence interval; IQR: Interquartile range; ORs: Odds ratios; UTI: Urinary tract infections; UTUC: Upper tract urothelial cancer. * Joint Wald tests performed for categorical variables.

## Data Availability

The data presented in this study are available on request from the corresponding author. The data are not publicly available due to prior approval necessary from CPRD.

## Supplementary Materials

The following are available online at https://www.mdpi.com/2072-6694/13/1/156/s1, Table S1: List of CPRD Medcodes used to define clinical features, Table S2: Sample derivation flowchart, Table S3: Association between patient and cancer characteristics and time to diagnosis beyond 45, 60 and 90 days.
