# Supplementary material for: Prolonged Diagnostic Intervals as Marker of Missed Diagnostic Opportunities in Bladder and Kidney Cancer Patients with Alarm Features: A Longitudinal Linked Data Study
Source: Cancers (Basel). 2021 Jan 5;13(1):156. doi: 10.3390/cancers13010156 (PMC7796444; doi:10.3390/cancers13010156)
Supplement: Supplementary file 1 [file cancers-13-00156-s001.pdf]

S1: List of CPRD Medcodes used to define clinical features

Medcode	Description
**Visible haematuria**	
6901	Clot haematuria
7232	Frank haematuria
9651	Painless haematuria
35555	Urine: red - blood
**Non-visible haematuria**	
2784	Microscopic haematuria
13915	RBCs- red blood cells in urine
13919	Urine: trace non-haemol. blood
13929	Urine blood test = +++
13932	Urine: trace haemolysed blood
13934	Urine blood test = +
19792	Urine blood test = ++
29463	Urine microscopy:RBC's present
**Non-specific haematuria**	
507	Haematuria
6030	Haematuria - symptom
6234	Blood in urine - symptom
6659	Blood in urine - haematuria
7164	Recurrent benign haematuria syndrome
17060	Recurrent and persistent haematuria
19361	Traumatic haematuria
20357	Painful haematuria
44541	Recurrent and persistent haematuria, dense deposit disease
47228	Essential haematuria

**Urinary tract infections**	
150	Urinary tract infection, site not specified NOS
389	Cystitis
532	Dysuria
1289	Urinary tract infection, site not specified
1353	Recurrent cystitis
1572	Recurrent urinary tract infection
1899	Pyelonephritis unspecified
2546	Acute pyelonephritis
2602	Cystitis of pregnancy
2650	Chronic urinary tract infection
2939	Calculous pyelonephritis
2985	Recurrent UTI
3469	Chronic cystitis unspecified
4498	[D]Painful urination
5350	[D]Dysuria
7014	C/O - ureteric pain
7579	Suspected UTI
8433	Urethral pain
9378	Recurrent urinary tract infections
10295	Acute gonococcal cystitis
10515	Recurrent urinary tract infection
10857	Other specified cystitis
11315	Other chronic cystitis NOS
12484	Cystitis NOS
12570	Post operative urinary tract infection
14644	UTI - urinary tract infection in pregnancy
15074	Acute cystitis
15599	[D]Dysuria NOS
15787	Urethral and urinary tract disorders NOS
22682	Cystitis cystica
29497	Prostatocystitis
30068	Subacute cystitis
32787	Other chronic cystitis
34630	Other cystitis NOS
38572	Xanthogranulomatous pyelonephritis
38698	Acute pyelonephritis NOS
48908	Chronic gonococcal cystitis
49235	Tuberculous pyelonephritis
52859	[X]Painful micturition, unspecified
53122	[X]Psychogenic dysuria
53944	Pyelonephritis and pyonephrosis unspecified
55781	Psychogenic dysuria
59121	Unspecified pyelonephritis NOS
64482	Acute pyelonephritis without medullary necrosis
70189	[X]Other cystitis
72686	[X]Other chronic cystitis
95710	Pyelonephritis in diseases EC
97002	Urinary tract infection
583	Urgency of micturition
4103	Polyuria
7300	Suprapubic pain
8028	[D] Urgency of micturition
8904	Urine smell
15389	[D]Vesical tenesmus
16399	[D]Polyuria

19983	Urine smell NOS
20475	[D]Suprapubic pain
23662	[D]Micturition frequency and polyuria
23841	Urine smell abormal
27002	Urine smell fishy
32031	Urine smell ammoniacal
53830	Polyuria
63898	[D]Frequency of micturition or polyuria NOS

**Abdominal mass**	
3015	[D]Abdominal mass
5838	[D]Swelling, mass or lump within abdomen or pelvis
7073	[D]Abdominal lump
8731	O/E - abdominal mass palpated
16370	[D]Swelling, mass or lump within abdomen or pelvis NOS
20387	O/E - abdominal mass shape
20827	Right iliac fossa mass
21301	O/E-abdominal mass consistency
21575	O/E left lower abdominal mass
21684	O/E -abd.mass fills 1 quadrant
21686	O/E - abdominal mass - soft
24034	[D]Umbilical mass
24527	Epigastric mass
34238	Biopsy of abdominal mass
51626	O/E - abdominal mass - hard
53736	[D]Lump stomach
59025	O/E - abd.mass consistency NOS
59578	O/E - abd.mass movt.with resp.
63484	O/E - abd. mass fills abdomen
65538	O/E -abd.mass -irregular shape
67090	O/E -abd.mass-upper border def
68388	O/E -abd.mass -border def. NOS
68939	O/E - abd.mass moves with resp
69694	O/E -abd.mass-lower border def
72512	O/E - abd.mass still with resp
97889	O/E - abdominal mass-very hard
98951	O/E - abd.mass + respn. NOS
99958	O/E - abd.mass -border defined
3006	[D]Groin lump
11442	[D]Umbilical lump
16670	[D]Umbilical swelling
21443	[D]Groin mass

4800	[D]Abdominal swelling

**Nocturia**	
840	Nocturia
5579	[D]Nocturia
**Poor stream**	
5038	Micturition stream poor
9274	[D]Slowing of urinary stream
**Hesitancy**	
7487	Hesitancy
**Urinary retention**	
1052	Cannot pass urine - retention
3002	[D]Acute retention of urine
5039	[D]Chronic retention of urine
5375	[D]Clot retention of urine
8393	[D]Post operative retention of urine
28017	[D]Retention of urine
**Urinary incontinence**	
2213	Bladder: incontinent
3283	H/O: stress incontinence
3887	Incontinence of urine
5844	Nocturnal enuresis
6161	Stress incontinence - symptom
13421	Urge incontinence of urine
15918	[D]Incontinence of urine

**Urolithiasis/ loin pain**	
19	Renal calculus
22	Bladder stone
23	Renal stone
24	Percutaneous lithotripsy of renal calculus
25	Ureteric calculus
39	Renal calculus NOS
45	Bladder calculus
47	Ureteric stone
54	O/E: kidney stone
63	Kidney calculus
65	Renal stone - uric acid
112	Other nephroscopic lithotripsy of ureteric calculus
120	Percutaneous lithotripsy of calculus in gall bladder
125	Uric acid bladder stone

135	Oxalate bladder stone
137	Phosphate kidney stone
149	O/E: renal stone NOS
179	Bladder calculus NOS
184	Phosphate bladder stone
202	O/E-vesical uric acid calculus
239	Urinary calculus NOS
298	Urinary calculus
325	Calculus of kidney
390	O/E - bladder calculus
415	O/E: renal calculus
429	Calculus of kidney and ureter
432	Staghorn calculus
460	O/E - ureteric calculus
563	Nephrolithiasis NOS
592	O/E: uric acid renal calculus
629	Calculus of kidney with calculus of ureter
630	O/E: oxalate renal calculus
679	Uric acid nephrolithiasis
686	Lower urinary tract calculus NOS
691	O/E - urethral calculus
693	[X]Urolithiasis
696	Other lower urinary tract calculus
700	O/E: prostatic calculus
705	Lower urinary tract calculus
715	O/E: cystine renal calculus
728	Urinary calculus in schistosomiasis
786	O/E - bladder calculus NOS
817	O/E - vesical oxalate calculus
824	Other calculus in bladder
6	C/O - loin pain
8	Renal colic
10	Left flank pain
12	Ureteric colic
27	C/O - renal pain
35	Right flank pain
48	[D]Renal colic
49	[D]Ureteric colic
52	Renal colic, symptom
59	Flank pain
61	C/O - ureteric colic
91	[D]Renal colic, unspecified
92	[D]Loin pain
101	O/E - renal angle tenderness
139	[D]Renal colic NOS

**Biliary colic**	
232	Biliary colic
378	Biliary colic
398	Biliary colic symptom
405	[D]Right upper quadrant pain
425	Right upper quadrant pain
421	Iliac fossa pain
1181	Right iliac fossa pain
1336	[D]Groin pain
2781	C/O pelvic pain
2982	Left iliac fossa pain
9061	[D]Left lower quadrant pain
9811	[D] Pelvic pain
9920	[D]Pelvic and perineal pain
11647	O/E - abd. pain - R.iliac
16547	Other pelvic pain - female
**Pelvic pain**	
16806	[D]Pain in right iliac fossa
16868	[D]Pain in left iliac fossa
17223	O/E - iliac pain on palpation
19360	[D]Right lower quadrant pain
21583	O/E - abd. pain - L.iliac
22608	Lower abdominal pain
35876	O/E - guarding - R.iliac
50662	to other parts of lower abdomen
56085	O/E - guarding - L.iliac
3419	Groin sprain
4706	Bony pelvic pain
7248	[D] Perineal pain
23715	Ischiopubic synchondrosis
29400	C/O perineal pain

**Abdominal pain**	
177	Abdominal pain
290	Epigastric pain
542	[D]Epigastric pain
716	[D]Abdominal cramps
1763	[D]Abdominal pain
1976	Abdominal pain type
2056	[D]Abdominal colic
2234	[D]Recurrent acute abdominal pain

2383	Abdominal discomfort
2657	Abdomen feels distended
3338	[D]Abdominal pain NOS
3978	Upper abdominal pain
4617	Central abdominal pain
4771	[D]Umbilical pain
5691	Non-colicky abdominal pain
5782	O/E - abdomen tender
5960	Site of abdominal pain
7812	Colicky abdominal pain
8362	[D]Left upper quadrant pain
8436	[D]Upper abdominal pain
11070	General abdominal pain-symptom
15908	Appendicular colic
16402	[D]Abdominal tenderness
17636	O/E - umbilical pain on palp.
19283	[D]Nonspecific abdominal pain
20640	O/E - epigastric pain on palp.
23756	[D]Evening colic
24627	O/E - abd. pain - umbilical
24661	Generalised abdominal pain
28285	[D]Gas pain (abdominal)
29352	Abdominal wall pain
29567	Abdomen feels bloated
31062	[D]Other specified abdominal pain
50590	O/E - abdominal rigidity
62927	O/E - rebound - umbilical
62933	O/E - guarding - umbilical
70357	[D]Tympanites (abdominal)
73235	O/E - abdominal rigidity NOS

**Low back pain**	
153	Backache
154	Low back pain
557	Backache, unspecified
2786	Backache symptom
3324	Back pain without radiation NOS
3763	C/O - low back pain
4680	Spasm of back muscles
5023	Acute back pain - lumbar
5035	Injury of muscle + tendon of abdomen, lower back and pelvis
5476	Acute back pain - disc
5840	Acute back pain with sciatica
5916	Acute back pain - thoracic
5923	Acute back pain - unspecified
6472	Backache symptom NOS
6704	Back pain, unspecified
8241	Backache with radiation
10231	Chronic low back pain
12189	Mechanical low back pain
24796	Back pain worse on sneezing
31011	Inj/nerves+lumbar spinal cord/abdo,lower back+pelvis level

**Abdominal distension**	
4800	[D]Abdominal swelling
6229	Abdominal distension symptom
14880	[D]Abdominal distension, gaseous
36966	Abdomen feels swollen

**Fatigue**	
1147	[D]Tiredness
1404	Fatigue
1582	Nervous exhaustion
1688	[D]Fatigue
1816	Malaise - symptom
4546	Chronic fatigue syndrome
5658	[D]Malaise
5751	Tired all the time
5794	Tiredness symptom
5814	[D]Lassitude
6190	Postviral fatigue syndrome
6242	Fatigue - symptom
7235	Tired all the time
7529	CFS - Chronic fatigue syndrome
9127	Post-viral fatigue syndrome
9220	Exhaustion
9656	[X]Fatigue syndrome
9823	C/O - debility - malaise
17083	Excessive exertion exhaustion
17526	Maternal exhaustion
17736	Malaise/lethargy
22923	Heat exhaustion, unspecified
23932	[D]Malaise and fatigue NOS
24382	[D]Senile exhaustion

27877	PVFS - Postviral fatigue syn
29181	[D]Post polio exhaustion
29292	Tiredness symptom NOS
43550	Combat fatigue
44215	[D]Malaise and fatigue
70779	[X]Combat fatigue
97284	Moderate chronic fatigue syndrome
98512	Mild chronic fatigue syndrome
98734	Severe chronic fatigue syndrome

**Weight loss**	
654	Weight decreasing
3647	[D]Abnormal loss of weight
4663	Abnormal weight loss
5812	Abnormal weight loss - symptom
12398	Complaining of weight loss
24068	[D]Cachexia
26473	O/E - weight > 20% below ideal
29029	O/E -weight 10-20% below ideal
37937	Weight loss from baseline weight
53801	[D]Cachexia NOS

**Loss of appetite**	
1855	[D]Appetite loss
6607	Loss of appetite - symptom
13081	Reduced appetite
17203	[X]Psychogenic loss of appetite

**Fever/ night sweats**	
1740	Night sweats
96946	[D]Night sweats
764	Acute rheumatic fever
1020	[D]Fever NOS
2389	[D]Pyrexia of unknown origin
2717	O/E -pyrexia of unknown origin
5859	Feels hot/feverish
5892	O/E - fever
5927	H/O: rheumatic fever
6065	Fever symptoms
6086	Pyrexia symptoms
7118	O/E - pyrexia - ? cause
8950	Feverish cold
9093	Pyrexial cold
9716	Unspecified viral haemorrhagic fever
14744	O/E - fever - general
14840	Acute rheumatic fever NOS
16792	[D]Pyrexial convulsion
17722	O/E - fever - remittent
17989	[D]Fever of unknown origin
18907	Cough with fever
19939	Sweating fever
20119	[D]Hyperpyrexia NOS
20367	[D]Pyrexia of unknown origin NOS
21430	Relapsing fever
22444	[D]Persistent fever
23349	[D]Chills with fever
25311	O/E - hyperpyrexia-> 40.5 oCEL
26478	Malignant hyperpyrexia due to anaesthetic
27231	O/E - temperature elevated
31412	O/E - fever - intermittent
35761	O/E - fever - general NOS
44756	Rheumatic fever with heart involvement
46292	O/E - fever NOS
47246	Fever with sweating
48189	Rheumatic fever without heart involvement
49816	Spotted fevers
52524	FH: Malignant hyperpyrexia
53952	O/E - fever - acute rise
59942	Other specified acute rheumatic fever
63219	Arenaviral haemorrhagic fever
66310	O/E - fever - continuous
73259	O/E - fever - gradual rise
93546	Omsk haemorrhagic fever
96114	[X]Unspecified viral haemorrhagic fever

**Anaemia**	
739	Anaemia unspecified
797	Macrocytic anaemia of unspecified cause
882	Hypochromic - microcytic anaemia
1702	Normocytic anaemia due to unspecified cause
2452	Protein-deficiency anaemia
2464	Pernicious anaemia
2482	Vit B12 defic anaemia due to malabsorption with proteinuria
2743	Acute posthaemorrhagic anaemia

2813	Addison's anaemia
3326	Haemolytic anaemias
3818	Autoimmune haemolytic anaemias
3981	Macrocytic anaemia unspecified cause
4080	Folate-deficiency anaemia
4475	Megaloblastic anaemia
4670	Secondary anaemia NOS
4952	O/E - anaemic
5271	Vitamin B12 deficiency anaemia
6028	Vitamin B12 deficiency anaemia, unspecified
6816	Normocytic anaemia due to chronic blood loss
7841	Deficiency anaemias
8054	Deficiency anaemias NOS
9537	Other specified iron deficiency anaemia NOS
10506	[X]Megaloblastic anaemia NOS
10817	Refractory anaemia with sideroblasts
11961	Other deficiency anaemias
12176	Chronic anaemia
12824	O/E - pale
14698	Hereditary haemolytic anaemia NOS
15314	Acquired haemolytic anaemia NOS
15358	O/E - anaemia
15422	Aplastic anaemia
15658	Acquired aplastic anaemia
15913	O/E - clinically anaemic
15914	O/E - profoundly anaemic
15936	Sideroblastic anaemia
16052	Refractory Anaemia
16109	O/E - anaemia NOS
18137	Unspecified iron deficiency anaemia
18631	Haemolytic anaemias NOS
19130	Refractory anaemia, unspecified
19383	Sideropenic anaemia
19574	Anaemia of prematurity
19951	Achlorhydric anaemia
21127	Iron deficiency anaemia due to dietary causes
21723	Acquired aplastic anaemia NOS
22531	Glucose-6-phosphate dehydrogenase deficiency anaemia
22715	Combined B12 and folate deficiency anaemia
22890	Refractory anaemia without sideroblasts, so stated
23875	Refractory anaemia with excess of blasts
24870	Folic acid deficiency anaemia
25046	O/E - colour pale
25876	Other specified anaemias
26327	Other specified deficiency anaemias
27726	Iron deficiency anaemia due to chronic blood loss
27771	Acquired haemolytic anaemias
28768	Other specified anaemia NOS
31270	Other vitamin B12 deficiency anaemia NOS
31550	Secondary sideroblastic anaemia due to disease
32715	Hypoplastic anaemia due to drug or chemical substance
32953	Vitamin B12 deficiency anaemia due to dietary causes
33278	Leukoerythroblastic anaemia
33420	Other specified iron deficiency anaemia
34754	Fanconi's familial refractory anaemia
34934	Other specified anaemias
34953	Transient hypoplastic anaemia
35092	[X]Other vitamin B12 deficiency anaemias
35160	Other anaemias NOS
36634	Folate-deficiency anaemia due to dietary causes
37082	Folate-deficiency anaemia, drug induced
37539	Aplastic and other anaemias
38327	Microangiopathic haemolytic anaemia
39456	Hereditary haemolytic anaemias
39876	Autoimmune haemolytic anaemia NOS
39944	Primary cold-type haemolytic anaemia
39967	Other specified haemolytic anaemias
40750	Idiopathic hypochromic anaemia
41142	Idiopathic aplastic anaemia
41699	Acquired sideroblastic anaemia
42117	Folate-deficiency anaemia NOS
43074	O/E - equivocally anaemic
43367	Mechanical haemolytic anaemia
43825	Normocytic anaemia due to aplasia
44420	Refractory anaemia with excess of blasts with transformation

44913	Hypoplastic anaemia - familial
45929	Normocytic anaemia following acute bleed
47225	Secondary sideroblastic anaemia due to drugs and toxins
47952	[X]Other dietary vitamin B12 deficiency anaemia
48338	Iron deficiency anaemia due to blood loss
49182	Primary warm-type haemolytic anaemia
50495	Acquired haemolytic anaemia with haemoglobinuria NEC
51169	[X]Other specified anaemias
51489	Other specified megaloblastic anaemia NEC
53783	[X]Other folate deficiency anaemias
53799	Megaloblastic anaemia NOS
53846	[X]Vitamin B12 deficiency anaemia, unspecified
55370	Biermer's congenital pernicious anaemia
55481	Folate-deficiency anaemia due to malabsorption
56348	Other specified megaloblastic anaemia NEC NOS
56756	[M] Refractory anaemia with sideroblasts
56973	Myasthenic syndrome due to pernicious anaemia
57114	Hypoplastic anaemia due to toxic cause
57274	Other deficiency anaemias NOS
57575	Secondary cold-type haemolytic anaemia
57859	Aplastic anaemia due to infection
57897	Non-autoimmune haemolytic anaemia
57954	Chlorotic anaemia
58136	Other specified nutritional deficiency anaemia
58695	Other specified other anaemia
59103	Megaloblastic anaemia due to dietary causes
60186	[M]Refractory anaemia+excess of blasts with transformation
60923	Disorder of iron metabolism NOS
62257	Vitamin E deficiency anaemia
63936	Infective haemolytic anaemia
64601	Amino-acid deficiency anaemia
64625	Fanconi's hypoplastic anaemia

65351	Hypoplastic anaemia due to infection
66137	Mediterranean anaemia
66239	Aplastic anaemia due to toxic cause
68087	Aplastic anaemia NOS
69027	Constitutional aplastic anaemia
69061	Constitutional aplastic anaemia without malformation
69275	Vegan's anaemia
69379	Other specified constitutional aplastic anaemia
70248	O/E: blood looks pale
70835	Protein-deficiency anaemia NOS
71840	[X]Nutritional anaemias
72276	Pyridoxine-responsive sideroblastic anaemia
72721	Other specified hereditary haemolytic anaemias
92106	Other specified nutritional deficiency anaemia NOS
94214	Non-autoimmune haemolytic anaemia NOS
94387	Sideroblastic anaemia NOS
94528	Asiderotic anaemia
94921	[M] Refractory anaemia with excess of blasts
98709	Goat's milk anaemia
99308	Other specified non-autoimmune haemolytic anaemia
99917	[X]Haemolytic anaemias
539	Microcytic - hypochromic anaemia
795	Iron deficiency anaemias
3265	Other and unspecified anaemias
4839	Microcytic hypochromic anaemia
15439	Iron deficiency anaemia NOS
29486	Other vitamin B12 deficiency anaemias

S2: Sample derivation flowchart

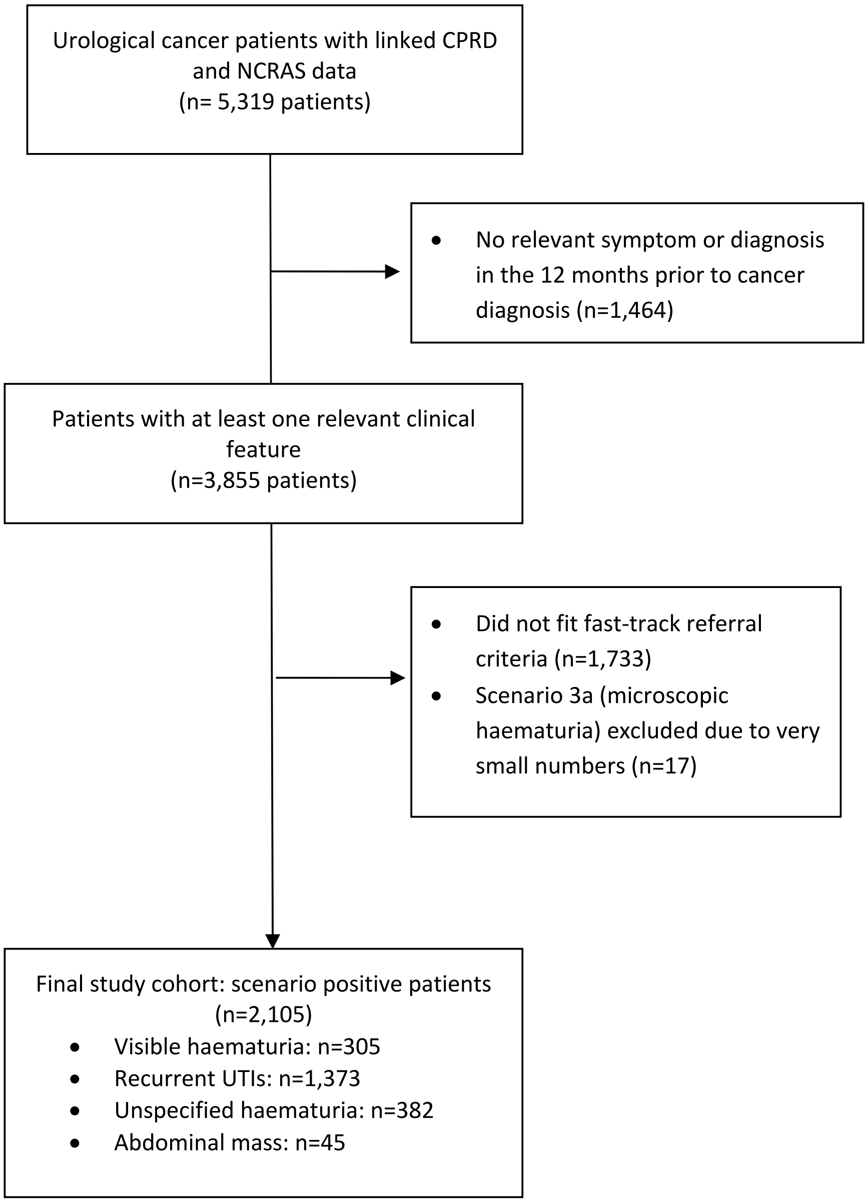

S3: Association between patient and cancer characteristics and time to diagnosis beyond 45, 60 and 90 days

	Adjusted Odds Ratio for Non-timely Diagnosis (95% CI)					
	**Beyond 45 days**	**p-value***	**Beyond 60 days**	**p-value***	**Beyond 90 days**	**p-value***
**Cancer Site**						
**Bladder**	Reference	<0.0001	Reference	<0.0001	Reference	<0.0001
**Kidney**	2.32 (1.74, 3.09)		2.28 (1.75, 2.96)		2.00 (1.52, 2.63)	
**UTUC**	3.19 (1.82, 5.58)		3.85 (2.32, 6.39)		2.75 (1.72, 4.38)	
**Scenario**						
**Visible haematuria**	Reference	<0.0001	Reference	<0.0001	Reference	<0.0001
**Recurrent UTIs**	2.08 (1.50, 2.89)		2.50 (1.81, 3.45)		3.46 (2.40, 5.00)	
**Unspecified haematuria**	1.13 (0.88, 1.45)		1.19 (0.92, 1.55)		1.43 (1.04, 1.98)	
**Abdominal mass**	0.67 (0.34, 1.30)		0.92 (0.47, 1.78)		1.03 (0.49, 2.20)	
**Sex**						
**Male**	Reference	0.1152	Reference	0.0292	Reference	0.0112
**Female**	1.18 (0.96, 1.46)		1.25 (1.02, 1.54)		1.33 (1.07, 1.66)	
**Age group**						
**<35**	1.03 (0.22, 4.78)	0.2200	1.05 (0.22, 4.87)	0.2009	1.31 (0.24, 7.04)	0.0471
**35-44**	1.42 (0.65, 3.09)		1.07 (0.52, 2.22)		0.75 (0.32, 1.73)	
**45-54**	0.88 (0.60, 1.28)		0.71 (0.49, 1.04)		0.48 (0.30, 0.77)	
**55-64**	0.91 (0.69, 1.19)		0.84 (0.64, 1.11)		0.83 (0.61, 1.14)	
**65-74**	Reference		Reference		Reference	
**75-84**	1.11 (0.89, 1.39)		1.09 (0.87, 1.35)		1.05 (0.82, 1.34)	
**85+**	1.39 (1.02, 1.91)		1.19 (0.88, 1.61)		1.06 (0.76, 1.48)	

CI: Confidence interval; UTIs: Urinary tract infections; UTUC: Upper tract urothelial cancer

** Joint Wald test performed for categorical variables*
